# Combined Effect of Microneedling and Platelet-Rich Plasma for the Treatment of Acne Scars: A Meta-Analysis

**DOI:** 10.3389/fmed.2021.788754

**Published:** 2022-02-14

**Authors:** Cheng Kang, Dakai Lu

**Affiliations:** Department of Otolaryngology-Head and Neck Surgery, HuaMei Hospital, University of Chinese Academy of Sciences, Ningbo, China

**Keywords:** acne scar, microneedling treatment, platelet-rich plasma, controlled studies, meta-analysis

## Abstract

**Background:**

Microneedling is a promising method for the treatment of acne scars, while the effect of microneedling combined with platelet-rich plasma (PRP) remains unknown. We performed a meta-analysis of controlled studies to compare the efficacy and safety of microneedling treatment with and without additional PRP in patients with acne scars.

**Methods:**

Randomized and non-randomized controlled studies were identified by search of Medline, Embase, and Cochrane's Library databases. Results were pooled with a random-effects model, incorporating the possible heterogeneity.

**Results:**

Four randomized and 10 split-face non-randomized controlled studies with 472 patients were included. Compared to microneedling therapy without PRP, combined treatment with microneedling and PRP was associated with increased odds of clinical improvement of >50% in Goodman's qualitative scale [GQS: odds ratio (OR): 2.97, 95% confidence interval (CI): 1.96–4.51, *p* < 0.001; *I*^2^ = 0%], and a significantly improved mean GQS score (mean difference: −0.32, 95% CI: −0.44 to −0.20, *p* < 0.001; *I*^2^ = 0%). Combined treatment was associated with a higher patient satisfying rate (OR: 4.15, 95% CI: 2.13 to 8.09, *p* < 0.001; *I*^2^ = 53%), while the incidence of severe adverse events such as severe erythema (OR: 1.59, 95% CI:.73 to 3.46, *P* = 0.24; *I*^2^ = 0%) and severe edema (OR: 1.14, 95% CI: 0.47 to 2.76, *P* = 0.77; *I*^2^ = 0%) were not significantly different.

**Conclusions:**

Combined treatment with microneedling with PRP is more effective than microneedling without PRP for patients with acne scars.

## Introduction

Acne vulgaris is a chronic inflammatory disease of skins, which mainly involves the hair follicles and sebaceous glands (1, 2). Acne is common, which could affect up to 80% of the adolescents (3, 4). The most common sequela of acne is acne scars, which could be formed in about 50% of people with acne, most commonly in highly visible areas such as faces and cheeks (5). Atrophic acne scars are the most common type of acne scars, which significantly affect the beauty of the patients and also impair the quality of life for some patients (6, 7). The pathogenesis of atrophic acne scars is complicated, which mainly involves the degradation of inflammatory mediators, collagen fibers, and subcutaneous fat, ultimately leading to the change of subcutaneous collagen deposition (8, 9). Multiple treatment strategies have been developed for the clinical management of acne scars (10, 11). Among them, microneedling is well-applied as an effective and minimally invasive technology for the treatment of atrophic acne scars *via* creation of skin microwounds to induce collagen production and dermal remodeling (12, 13). Besides, platelet-rich plasma (PRP), which contains various growth factors and bioactive cytokines, has also been applied as adjuvant therapy for acne scars (14, 15). Some small-scale clinical studies have observed the role of combined treatment with microneedling and PRP for patients with atrophic acne scars (16–29). However, the efficacy and safety of microneedling treatment combined with PRP in patients with atrophic acne scars have rarely been comprehensively evaluated. Accordingly, we performed a meta-analysis of controlled studies to systematically compare the efficacy and safety of microneedling treatment with and without additional PRP in patients with acne scars.

## Methods

The PRISMA (Preferred Reporting Items for Systematic Reviews and Meta-Analyses) statement (30) and the Cochrane Handbook guidelines (31) were followed during the designing and implementation of the study.

### Search Strategy

Medline, Embase, and the Cochrane's Library (Cochrane Center Register of Controlled Trials) databases were searched for relevant studies with a combined strategy of: (1) “platelet-rich plasma” OR “PRP” OR “platelet-rich plasma” OR “platelet concentrate” OR “platelet concentrates”; (2) “needle” OR “microneedle” OR “microneedling”; and (3) “scar” OR “scars.” This expanded search strategy was used to avoid missing possible relevant studies. Only clinical studies in humans and published in English were considered. The references of related reviews and original articles were also searched as complementation. The final database search was conducted on June 10, 2021.

### Study Selection

Studies that fulfilled the following criteria were included: (1) articles published as full-length articles in English; (2) designed as split-fact non-randomized controlled studies or parallel-group randomized controlled trials (RCTs); (3) included patients with atrophic acne scars allocated to an interventional group with microneedling and PRP and a control group with microneedling without PRP; and (4) reported at least one of the following outcomes, including the rate of significant clinical improvement defined as >50% improvement in Goodman's qualitative scale (GQS), changes of mean GQS from the baseline, the rate of patient satisfaction, and incidence of severe adverse events. The clinical improvement was assessed by the dermatologists based on GQS. Reviews, studies not including patients with atrophic acne scars, without interventions of microneedling or PRP, without a control group, or studies that did not report the outcomes of interest were excluded.

### Data Extraction and Quality Assessment

Database search, data extraction, and quality evaluation were conducted by two independent authors. If disagreement occurred, it was resolved by consensus between the authors. We extracted data on study information (first author, publication year, and study country), study design (randomized or non-randomized), patient information (number of participants, the range of age, and sex), intervention and control (details of microneedling applied, PRP preparation, and sessions), follow-up duration, and outcomes reported. For RCTs, quality evaluation was achieved using the Cochrane's Risk of Bias Tool (31) according to the following aspects: (1) random sequence generation; (2) allocation concealment; (3) blinding of participants and personnel; (4) blinding of outcome assessors; (5) incomplete outcome data; (6) selective outcome reporting; and (7) other potential bias. For non-randomized studies, quality evaluation was performed with the Newcastle—Ottawa Scale (NOS) (32), which included three domains, such as defining of study groups, between-group comparability, and validation of the outcome. The NOS totally scored from 1 to 9 stars, with 9 stars indicating the highest study quality level.

### Statistical Analysis

Incidence of significant clinical improvement, patient satisfaction, and adverse events were separately evaluated *via* odds ratios (ORs) and their 95% confidence intervals (CIs) in this meta-analysis, while changes of mean GQS from the baseline were evaluated with mean difference (MD) and corresponding 95% CI. We used Cochrane's Q test to detect the heterogeneity (33). The *I*^2^ statistic was also calculated, and an *I*^2^ > 50% reflected significant heterogeneity. Pooled analyses were calculated using a random-effects model because this method incorporates the influence of potential heterogeneity and retrieves a more generalized result (31). Sensitivity analysis was performed to compare the treatments between microneedling and PRP with microneedling alone (31). Predefined subgroup analyses were used to evaluate the possible influences of study design on the outcomes. Publication bias was evaluated by visual inspection of funnel plots, and Egger's regression asymmetry test (34). *P* < 0.05 were considered statistically significant. The RevMan (Version 5.1; Cochrane, Oxford, UK) and Stata software (Version 12.0; Stata, College Station, TX) were applied for statistical analyses.

## Results

### Search Results

**Figure 1:**
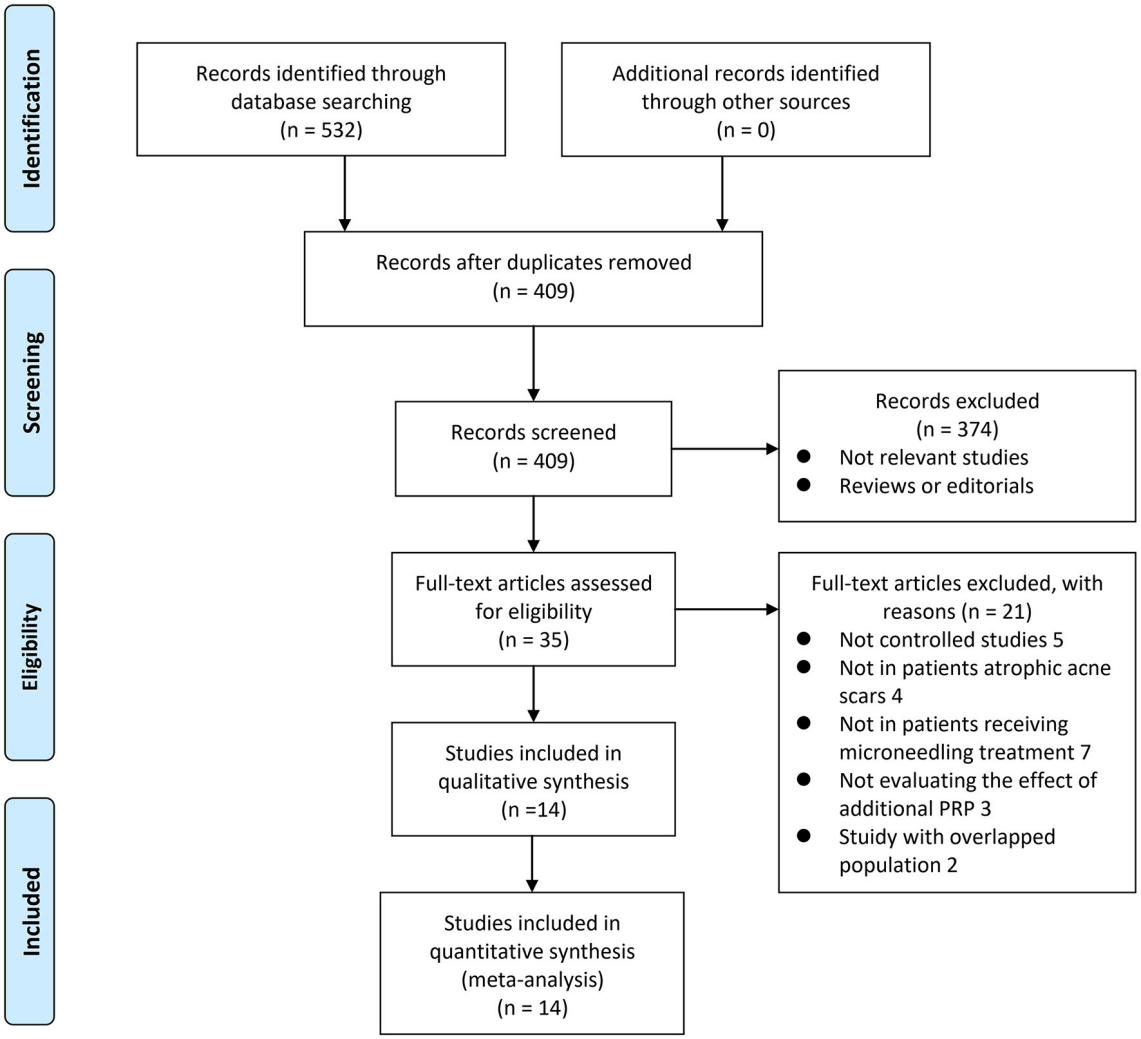
A flow chart of literature search. From Moher et al. (35).

The process of database search and study identification is shown in Figure 1. Briefly, 532 articles were obtained through the database search, and 409 were retrieved after exclusion of duplicated records. Among them, 374 articles were subsequently excluded based on titles and abstracts, primarily because these studies were irrelevant to the aim of the meta-analysis. Of the 35 articles that underwent full-text review, 21 were further excluded for the reasons presented in Figure 1. Finally, 14 studies, including four RCTs (19, 22–24) and 10 non-randomized controlled studies (16–18, 20, 21, 25–29) were included.

### Study Characteristics

Table 1 shows the characteristics of the included studies. Overall, four RCTs (19, 22–24) and 10 face-split non-randomized controlled studies (16–18, 20, 21, 25–29) with 472 patients were included in the meta-analysis. The studies were published between 2011 and 2021. The studies were mostly performed in India (17, 18, 22, 23, 26–29) and Egypt (19–21, 24, 25), as well as one study in Italy (16). All of the studies included patients with acne scars. The mean ages varied between 23 and 33 years, and the proportions of men ranged between 25 and 70%. The details of PRP preparation are also summarized in Table 1, which typically included two steps of centrifugation of the autologous whole blood, except for one study (27), which applied a one-step centrifugation method. For the comparisons, interventions of combined microneedling and PRP were compared with controls of microneedling alone in nine studies (16, 19–24, 26, 27), with microneedling and distilled water or normal saline in two studies (18, 29), and with microneedling and vitamin C, hyaluronic acid, and insulin in the other three studies (17, 25, 28). For each treatment, two to six sessions were applied, with the follow-up durations varying from 4 to 32 weeks.

**Table 1:** Characteristics of the included studies.

**References**	**Country**	**Design**	**No. of patients**	**Scar characteristics**	**Age (years)**	**Male (%)**	**Details of MN**	**PRP preparation**	**Intervention**	**Control**	**Sessions**	**Follow-up duration (weeks)**	**Outcome assessment**
Fabbrocini et al. (16)	Italy	NRCT, Split-face study	12	Acne scars	32.2	41.7	Using rolling barrel 1-mm wide, equipped with 96 needles in 4 rows	70–80 ml autologous whole blood centrifuged at 1,200 g for 15 min, then further centrifuged at 1,800 g for 10 min, to get a 4.5 × PRP	MN + PRP	MN only	2	32	Rate of GQS improvement and changes of GQS
Chawla (17)	India	NRCT, Split-face study	27	Grade 2–4 acne scars	27.5	70	Using rolling drum of 192 needles	10 ml autologous whole blood centrifuged at 1500 rpm for 10 min, then further centrifuged at 3700 rpm for 10 min, to get a 4.5x PRP	MN + PRP	MN + Vitamin C	3	4	Rate of GQS improvement and patient satisfying
Asif et al. (18)	India	NRCT, Split-face study	50	Grade 2–4 acne scars	25.7	50	Using a standard roller device with 192 needles, each of 1.5 mm in size	17 ml autologous whole blood centrifuged at 294 g for 5 min, then further centrifuged at 691 g for 17 min, to get a 5.2 × PRP	MN + PRP	MN + distilled water	3	12	Rate of GQS improvement and patient satisfying
Ibrahim et al. (19)	Egypt	RCT, OL	36	Acne scars	26.3	49	Using an automated microneedling device (dermapen 3) with nine microneedles of 0.25 mm to 2.5 mm length	20 ml autologous whole blood centrifuged at 1,419 g for 7 min, then further centrifuged at 2,522 g for 5 min, to get a 10 × PRP	MN + PRP	MN only	6	12	Rate of GQS improvement and incidence of severe side effect
Porwal et al. (22)	India	RCT, OL	55	Grade 2–4 acne scars	26	46.2	Using a dermaroller with 192 needles of 1.5 mm length	20 ml autologous whole blood centrifuged at 1,200 rpm for 15 min, then further centrifuged at 2,000 rpm for 10 min, to get a PRP	MN + PRP	MN only	3	4	Changes of GQS and incidence of severe side effect
El-Domyati et al. (20)	Egypt	NRCT, Split-face study	24	Acne scars	27.3	25	Using a dermaroller with 600 needles of 1.5 mm length	10 ml autologous whole blood centrifuged at 252 g for 10 min, then further centrifuged at 1,792 g for 5 min, to get a PRP	MN + PRP	MN only	2	12	Rate of GQS improvement
Ibrahim et al. (21)	Egypt	NRCT, Split-face study	35	Acne scars	24.7	NR	Using a dermaroller with 192 needles of 1.5 mm length	10 ml autologous whole blood centrifuged at 2,500 rpm for 10 min, then further centrifuged at 3,500 rpm for 10 min, to get a PRP	MN + PRP	MN only	4	12	Changes of GQS and rate of patient satisfying
Bhargava et al. (23)	India	RCT, SB	30	Grade 4 acne scars	27.7	36.7	Using a dermaroller with 192 needles of 1.5 mm length	10 ml autologous whole blood centrifuged at 1,500 rpm for 10 min, then further centrifuged at 3,700 rpm for 10 min, to get a 4.5 × PRP	MN + PRP	MN only	3	12	Rate of GQS improvement and patient satisfying
Elfar and Hasby (24)	Egypt	RCT, SB	40	Grade 2–4 acne scars	27.2	30	Using a dermaroller with 540 needles of 2.0 mm length	10 ml autologous whole blood centrifuged at 72 g for 15 min, then further centrifuged at 1,006 g for 5 min, to get a PRP	MN + PRP	MN only	4	12	Rate of GQS improvement
Sharma et al. (29)	India	NRCT, Split-face study	40	Grade 2–4 acne scars	25.3	32.5	Using a dermaroller with 192 needles of 1.5 mm length	10 ml autologous whole blood centrifuged at 1,500 rpm for 10 min, then further centrifuged at 3,700 rpm for 10 min, to get a 4.5 × PRP	MN + PRP	MN + NS	4	24	Rate of GQS improvement, changes of GQS, and incidence of severe side effect
Amer et al. (25)	Egypt	NRCT, Split-face study	41	Grade 2–4 acne scars	28	31.7	Using a dermaroller	10 ml autologous whole blood centrifuged at 1,600 rpm for 10 min, then further centrifuged at 4,000 rpm for 15 min, to get a PRP	MN + PRP	MN + HA	4	4	Rate of GQS improvement and patient satisfying
Pawar and Singh (28)	India	NRCT, Split-face study	16	Acne scars	24.7	43.8	Using a dermaroller with 192 needles of 1.5 mm length	10 ml autologous whole blood centrifuged at 1,500 rpm for 10 min, then further centrifuged at 3,700 rpm for 10 min, to get a 4.5 × PRP	MN + PRP	MN + insulin	4	12	Changes of GQS and incidence of severe side effect
Gupta et al. (26)	India	NRCT, Split-face study	36	Acne scars	23.7	47.2	Using a dermaroller with 192 needles of 2.0 mm length	8.5 ml autologous whole blood centrifuged at 1,400 rpm for 10 min, then further centrifuged at 3,500 rpm for 10 min, to get a PRP	MN + PRP	MN only	4	24	Changes of GQS
Nandini et al. (27)	India	NRCT, Split-face study	30	Grade 2–4 acne scars	25	60	Using a dermaroller with 192 needles of 2.0 mm length	6 ml autologous whole blood centrifuged at 3,600 rpm for 15 min to get a PRP	MN + PRP	MN only	4	12	Rate of GQS improvement and patient satisfying

*MN, microneedling; PRP, platelet-rich plasma; NRCT, nonrandomized controlled trials; RCT, randomized controlled trials; OL, open-label; SB, single-blind; HA, hyaluronic acid; GQS, Goodman's qualitative scale; NR, not reported; NS, normal saline*.

### Data Quality

Table 2 shows the details of the study quality of the included non-randomized studies (16–18, 20, 21, 25–29). The NOS varied between 8 and 9 stars, indicating generally good study quality. Table 3 shows the details of study quality of the included RCTs (19, 22–24). Two of the RCTs were open-label (19, 22), and the other two were single-blinded (23, 24). The method of random sequence generation and information of allocation concealment was reported in only one RCT (24). The overall quality score varied between 3 and 7, indicating moderate to good study quality.

**Table 2:** Quality evaluation *via* the Newcastle—Ottawa Scale for non-randomized controlled studies.

**References**	**Representativeness of the exposed cohort**	**Selection of the non-exposed cohort**	**Ascertainment of exposure**	**Demonstration that outcome of interest was not present at start of study**	**Comparability-age and gender**	**Comparability-other factors**	**Assessment of outcome**	**Was follow-up long enough for outcomes to occur**	**Adequacy of follow-up of cohorts**	**Total**
Fabbrocini et al. (16)	1	1	1	1	1	1	1	1	1	9
Chawla (17)	1	1	1	1	1	1	1	1	0	8
Asif et al. (18)	1	1	1	1	1	1	1	1	1	9
El-Domyati et al. (20)	1	1	1	1	1	1	1	1	0	8
Ibrahim et al. (21)	1	1	1	1	1	1	1	1	1	9
Sharma et al. (29)	1	1	1	1	1	1	1	1	1	9
Amer et al. (25)	1	1	1	1	1	1	1	1	1	9
Pawar and Singh (28)	1	1	1	1	1	1	1	1	0	8
Gupta et al. (26)	1	1	1	1	1	1	1	1	0	8
Nandini et al. (27)	1	1	1	1	1	1	1	1	1	9

**Table 3:** Quality evaluation for the randomized controlled trials *via* the Cochrane's Risk of Bias Tool.

**References**	**Random sequence generation**	**Allocation concealment**	**Blinding of participants**	**Blinding of outcome assessment**	**Incomplete outcome data addressed**	**Selective reporting**	**Other sources of bias**	**Total**
Ibrahim et al. (19)	Unclear	Unclear	High	High	Low	Low	Low	3
Porwal et al. (22)	Unclear	Unclear	High	High	Low	Low	Low	3
Bhargava et al. (23)	Unclear	Unclear	High	Low	Low	Low	Low	4
Elfar and Hasby (24)	Low	Low	High	Low	Low	Low	Low	6

### Meta-Analysis Results

**Figure 2:**
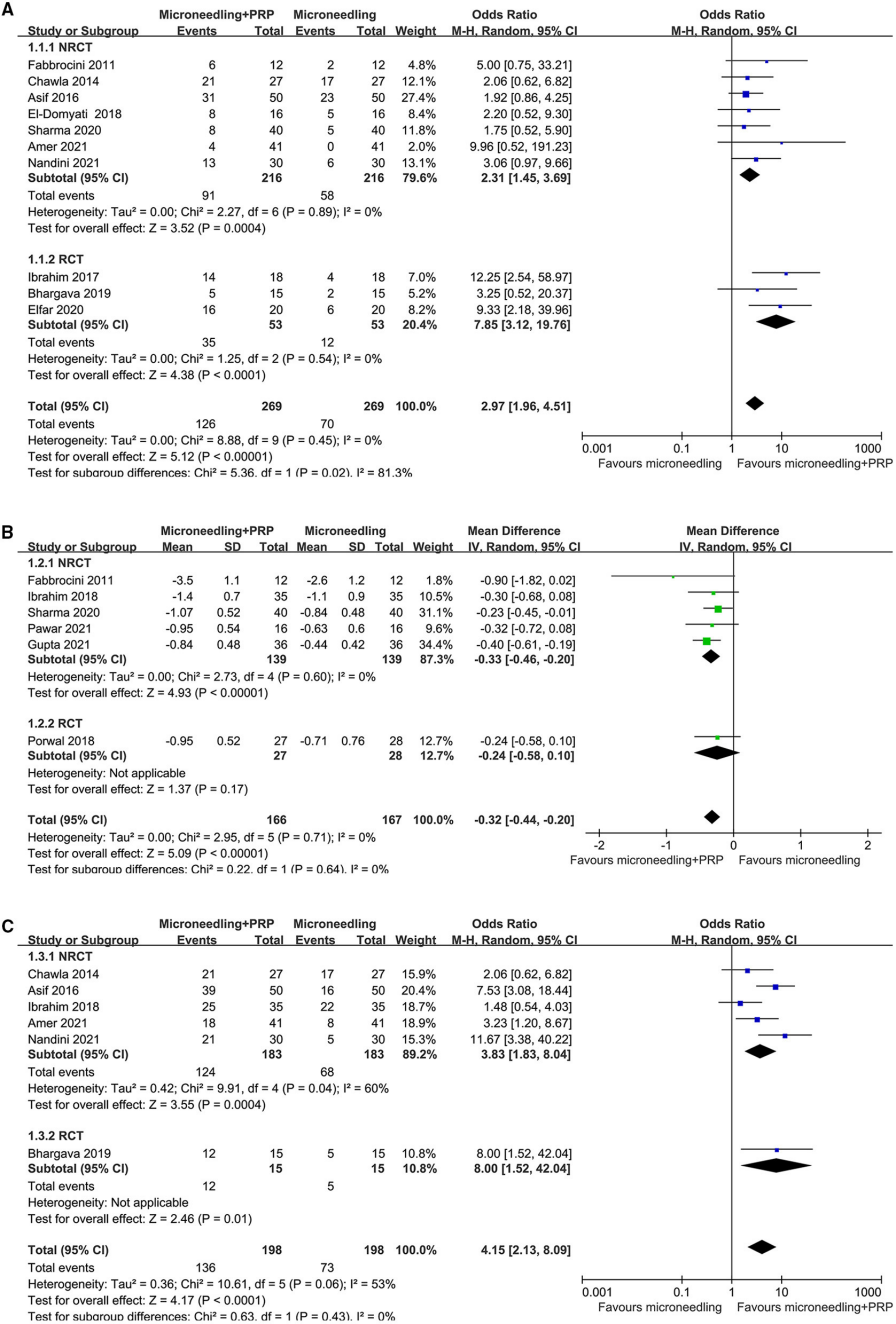
Forest plots for the meta-analyses, comparing the effect of combined treatment with microneedling and PRP vs. microneedling without PRP for patients with acne scars; **(A)** the rate of significant clinical improvement; **(B)** difference of changes of GQS after treatment; and **(C)** the rate of patient satisfaction.

**Figure 3:**
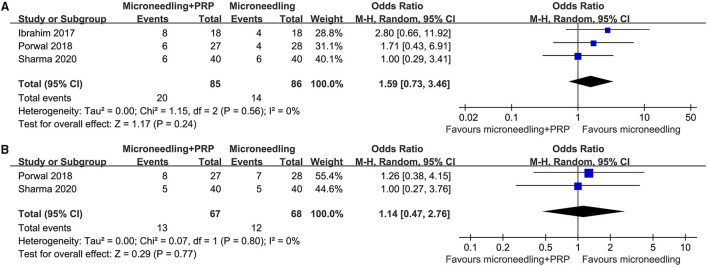
Forest plots for the meta-analyses, comparing the safety of combined treatment with microneedling and PRP vs. microneedling without PRP for patients with acne scars; **(A)** incidence of severe erythema; and **(B)** incidence of severe edema.

Pooled results of 10 studies (16–20, 23–25, 27, 29) showed that combined treatment with microneedling and PRP was associated with increased odds of significant clinical improvement as compared with microneedling therapy without PRP (OR: 2.97, 95% CI: 1.96 to 4.51, *p* < 0.001; Figure 2A) without significant heterogeneity (*P* for Cochrane's *Q*-test = 0.45, *I*^2^ = 0%). Sensitivity analysis showed consistent results limited to the comparisons between the combined treatment and microneedling alone (OR: 4.58, 95% CI: 2.49 to 8.42, *p* < 0.001; *I*^2^ = 0%). Subgroup analyses according to the study design showed both significant results in non-randomized studies and RCTs, while the effect was more remarkable in RCTs than in non-randomized studies (OR: 7.85 vs. 3.02, P for subgroup difference = 0.02; Figure 2A). In addition, combined treatment with microneedling and PRP was associated with a significantly improved mean GQS score (six studies MD: −0.32, 95% CI: −0.44 to −0.20, *p* < 0.001; *I*^2^ = 0%; Figure 2B) and a higher patient satisfying rate (six studies, OR: 4.15, 95% CI: 2.13 to 8.09, *p* < 0.001; *I*^2^ = 53%; Figure 2C) compared to treatment with microneedling without PRP. Subgroup analyses showed that difference in study design did not significantly affect the results (*P* for subgroup difference = 0.22 and 0.63, respectively). Meta-analyses of three and two studies showed that the incidence of severe adverse events such as severe erythema (OR: 1.59, 95% CI: 0.73 to 3.46, *P* = 0.24; *I*^2^ = 0%; Figure 3A) and severe edema (OR: 1.14, 95% CI: 0.47 to 2.76, *P* = 0.77; *I*^2^ = 0%; Figure 3B) were not significantly different between the two treatment groups.

### Publication Bias

**Figure 4:**
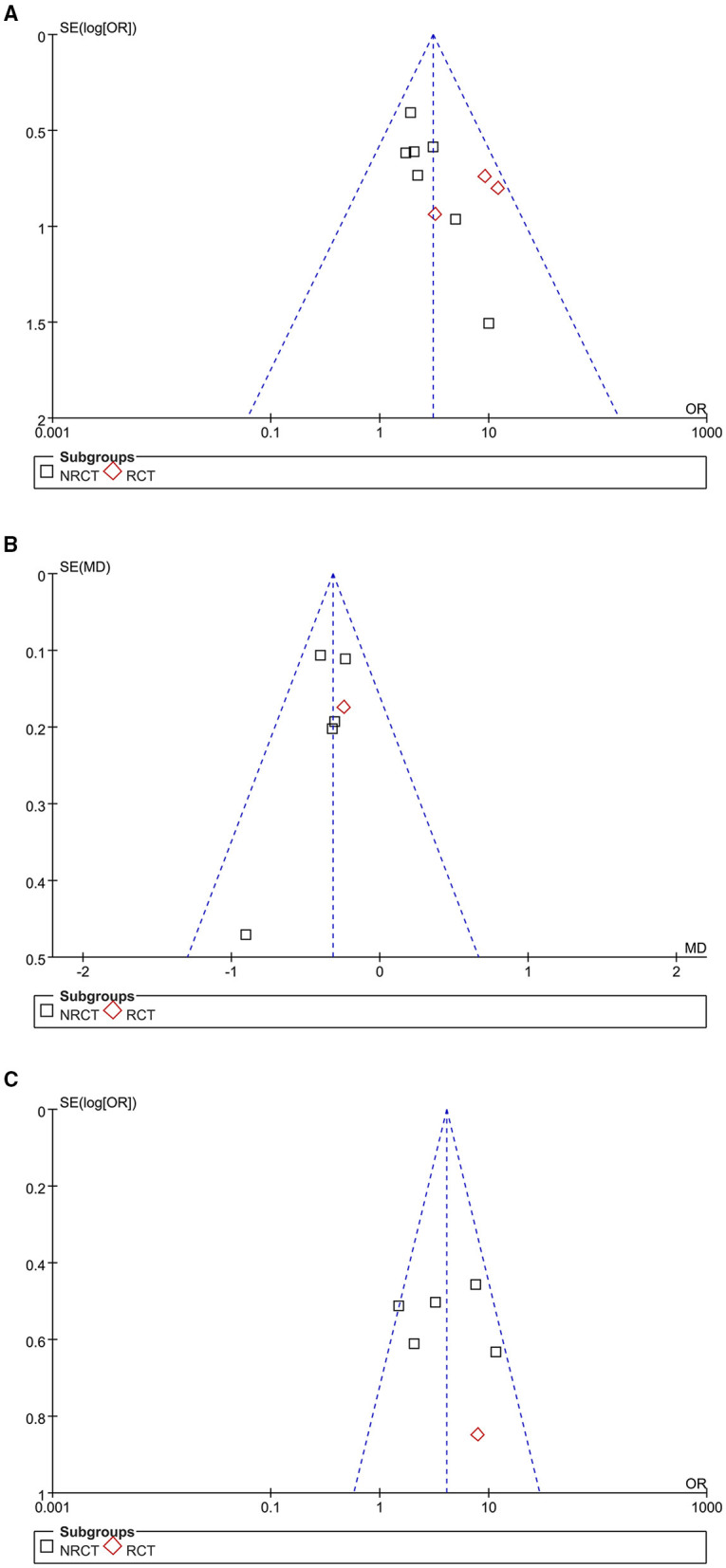
Funnel plots for the publication bias within the meta-analyses, comparing the effect of combined treatment with microneedling and PRP vs. microneedling without PRP for patients with acne scars; **(A)** funnel plots for the rate of significant clinical improvement; **(B)** funnel plots for the difference of changes of GQS after treatment; and **(C)** funnel plots for the rate of patient satisfaction.

The funnel plots for the meta-analyses of outcomes of the significant clinical improvement rate, changes of GQS, and the patient satisfying rate are shown in Figures 4A–C, respectively. These plots were symmetrical, suggesting low risk of publication biases among the outcomes. Egger's regression tests also showed low risks of publication biases for these meta-analyses (*P* for Egger's regression test = 0.534, 0.328, and 0.297, respectively).

## Discussion

In this meta-analysis, by pooling the results of 14 non-randomized studies and RCTs, we found that a combined treatment with microneedling and PRP was associated with better improvement of atrophic acne scars than microneedling without PRP. The efficacy of a combined treatment was evidenced by the higher rate of significant improvement and better improved GQS evaluated by dermatologists and the higher satisfying rate from the patients. Besides, pooled results of limited datasets suggested no significant difference in the incidence of severe adverse events between groups, such as severe erythema and edema. Taken together, the results of our meta-analysis indicated that combined treatment with microneedling with PRP is more effective than microneedling without PRP for patients with acne scars, without further increasing the risk of adverse events. These findings support PRP as an adjuvant therapy for patients with acne scars receiving microneedling therapy.

To the best of our knowledge, few meta-analyses were performed to evaluate the efficacy and safety of additional PRP as an adjuvant therapy for patients with acne scars receiving microneedling therapy. A previous meta-analysis, including five comparative studies before 2019 with 226 patients with atrophic scars showed that a combined treatment with microneedling and PRP may confer better efficacy in patients with atrophic acne scars than treatment with microneedling alone. However, the results were based on limited studies, and other efficacy outcomes, such as changes of GQS, were unable to be determined (36). Our study has several strengths compared to the previous one (36). Firstly, an up-to-date and compressive literature search was performed. With the expanded literature search strategy, we identified 14 comparative studies, including 472 patients, which were much larger than the previous one. Moreover, the relatively larger number of studies enables us to evaluate the efficacy of a combined therapy with microneedling and PRP in a multidimensional manner, including the outcomes of the significant clinical improvement rate, between-group difference of GQS changes, and the patient satisfying rate. All of these outcomes consistently indicated a better efficacy of the combined therapy with PRP compared to microneedling without PRP for acne scars. In addition, safety outcomes were also explored, which did not show that additional use of PRP was associated with an increased risk of severe adverse events. Finally, sensitivity analysis and subgroup analysis according to the design of the studies were performed, and the consistent results further assured the stability of the findings.

The possible synergistic effect of PRP on the basis of microneedling treatment for atrophic acne scars may be explained by multiple mechanisms (37). The primary mechanisms for the microneedling treatment are the formation of skin microwounds to induce collagen production and dermal remodeling (12, 13). Previous studies have confirmed that PRP contains multiple active cytokines and growth factors that are primarily released from the a-granules of platelets and actively involved in the process of dermal remodeling, such as the dermal-transforming growth factor, the platelet-derived growth factor, the vascular endothelial growth factor, and the fibroblast growth factor (38, 39). The skin microwounds resulting from the microneedling therapy may also enhance the absorption of the above active cytokines in PRP by the skin (12, 13). However, studies evaluating the mechanisms underlying the synergetic therapeutic efficacy of microneedling and PRP are limited. Interestingly, a recent study has shown that, compared to the use of microneedling alone, combined treatment with microneedling and PRP was associated with a marked excellent improvement of the skin lesions, a more significant deposition of collagen and elastic fibers, and an increased proliferative activity in the epidermis in people with striae distensa, which may also reflect the synergetic benefits of action microneedling and PRP on scar formation and collagen/dermal remodeling (40). Interestingly, the efficacy of combined treatment with microneedling and PRP has also been observed in people with androgenetic alopecia (41) and melisma (42). The exact molecular mechanisms underlying the benefits of a combined therapy with microneedling and PRP for atrophic acne scars should be further evaluated.

Some limitations of the current meta-analysis should be considered when the results are interpreted. Firstly, the numbers of available studies and the included patients for some outcomes were limited. For example, meta-analysis of adverse events only involved 2–3 studies; the results of which should be validated in large-scale RCTs. In addition, the limited number of available datasets may also reduce the reliability of funnel plots for the estimation of publication biases. In particular, it has been suggested that visual interpretation of funnel plots is too subjective to be useful (31), and it has been pointed out that the researchers had only a limited ability to identify correctly funnel plots for meta-analyses that were subject to bias due to missing results (43). Moreover, quality scores of the included RCTs were moderate, and 10/14 of the included studies were non-randomized face-split studies, although subgroup analysis according to study design was performed. Accordingly, high-quality large-scale RCTs with adequate statistical power are warranted to verify our findings. In addition, the optimal PRP formulation remains unknown, and the influence of PRP formulation on the outcomes was not performed because most of the included studies did not provide adequate details related with PRP preparation. Finally, most of the studies were performed in India and Egypt. Studies from the other regions of the world are warranted to validate the findings.

In conclusion, the results of our meta-analysis indicated that combined treatment with microneedling with PRP is more effective than microneedling without PRP for patients with acne scars, without further increasing the risk of adverse events. Although large-scale RCTs are warranted to confirm these results, the findings of the meta-analysis support using PRP as an adjuvant therapy for patients with acne scars receiving microneedling therapy.

## Data Availability Statement

The original contributions presented in the study are included in the article/supplementary material, further inquiries can be directed to the corresponding author/s.

## Author Contributions

CK and DL designed the study, performed database search, data extraction, statistical analyses, and interpretation of the results. CK drafted the manuscript. DL critically revised the manuscript. All authors approved the submission of the manuscript.

## Funding

This study was supported by HwaMei Research Foundation of HwaMei Hospital and University of Chinese Academy of Sciences (No. 2022HMZD10).

## Conflict of Interest

The authors declare that the research was conducted in the absence of any commercial or financial relationships that could be construed as a potential conflict of interest.

## Publisher's Note

All claims expressed in this article are solely those of the authors and do not necessarily represent those of their affiliated organizations, or those of the publisher, the editors and the reviewers. Any product that may be evaluated in this article, or claim that may be made by its manufacturer, is not guaranteed or endorsed by the publisher.
